# Association of DNA repair gene polymorphisms with the risk of radiation pneumonitis in lung cancer patients

**DOI:** 10.18632/oncotarget.22982

**Published:** 2017-12-05

**Authors:** Lehui Du, Wei Yu, Xiangkun Dai, Nana Zhao, Xiang Huang, Fang Tong, Fang Liu, Yurong Huang, Zhongjian Ju, Wei Yang, Xiaohu Cong, Chuanbin Xie, Xiaoliang Liu, Lanqing Liang, Yanan Han, Baolin Qu

**Affiliations:** ^1^ Department of Radiation Oncology, Chinese PLA General Hospital, Beijing 100853, China

**Keywords:** radiation pneumonitis, ERCC1, ERCC2, XRCC1, lung cancer

## Abstract

A total of 149 lung cancer patients were recruited to receive intensity modulated radiation therapy (IMRT). The association of developing radiation pneumonitis (RP) with genetic polymorphism was evaluated. The risks of four polymorphic sites in three DNA repair related genes (ERCC1, rs116615:T354C and rs3212986:C1516A; ERCC2, rs13181:A2251C; XRCC1, rs25487:A1196G) for developing grade ≥ 2 RP were assessed respectively. It was observed that ERCC1 T354C SNP had a significant effect on the development of grade ≥ 2 RP (CT/TT vs. CC, adjusted HR = 0.517, 95% CI, 0.285–0.939; adjusted *P =* 0.030). It is the first time demonstrating that CT/TT genotype of ERCC1 354 was significantly associated with lower RP risk after radio therapy.

## INTRODUCTION

Radiation therapy currently plays a crucial role in the treatment of lung cancer, primarily because of both its radical and palliative cure [1]. However, radiation pneumonitis (RP), which has been identified as the most significant dose-limiting toxicity with 5%–36% of patients experiencing serve symptom (grade 2–5 RP) [2–3] is one of major obstacles for clinical application of radio therapy, and radiation pneumonitis even has been suggested by previous studies as an independent negative prognostic factor for the survival of lung cancer patients [4]. Many factors, including age, sex, smoking, radiation dose, irradiated lung volume, surgery and chemotherapy, are often used to estimate RP risk [5–7]. Currently in clinical use, dosimetric parameters, such as the volume of the lung that received 20 Gy radiation dose (V20) and median lung dose (MLD) are important determinants of radiotherapy-induced lung toxicity [8–10]. Unfortunately, consideration of only these factors is definitely insufficient to evaluate the risk of developing RP, and several relative studies have suggested that RP has a genetic basis [11–13]. Currently, there is an increasing consensus that RP is considered as a so-called complex, inherited polygenic trait. Single-nucleotide polymorphisms (SNPs) have been proposed as a potential predictive biomarker for the development of RP. SNPs in inflammation, DNA repair, stress response and angiogenesis-related genes were proved to be associated with RP, with different underlying mechanisms [14].

Radiotherapy exerts its effects on damaging cells primarily either directly, by attacking cellular macromolecules, including genomic DNA, or indirectly, by generating reactive oxygen species and subsequent byproducts. Both types of mechanisms result in extensive DNA damage and cell-death [15]. Hence, repair of radiation-induced DNA damage is considered as one of the most important cellular defense mechanisms against radiation pressure [16]. SNPs may change protein total level and/or functional activities and thereby impact DNA repair capacity [17]. To date, there are four major pathways have been discovered for repairing DNA lesion, as follows: base excision repair (BER); nucleotide excision repair (NER); DNA double-strand break repair by homologous recombination (HR) and non-homologous end joining (NHEJ).

Nucleotide excision repair (NER) as one of the most important mechanisms for repairing has been used by cells to repair a wide variety of DNA structural lesions, including bulky adducts, cross-links, oxidative DNA damages, thymidine dimmers and alkylating damages [18]. Excision repair cross-complimentary group 1 and group 2 (ERCC1 and ERCC2), both located in the chromosome 19q13.2-13.3, are two key rate-limiting enzymes and are indispensable for NER. X-ray repair cross-complementing group 1 (XRCC1) belongs to BER system, which is responsible for the removal of single damaged DNA bases and for efficient repair of DNA single-strand breaks generated extensively by radiation therapy [19].

The aim of this study was to evaluate the possible relationship between several common genetic polymorphisms in DNA repair genes (ERCC1, ERCC2 and XRCC1) and the risk of developing RP in lung cancer patients receiving intensity modulation radiated therapy.

## RESULTS

### Patient characteristics and clinical outcomes

Table 1 displays the characteristics of 149 lung cancer patients including 127 (85.2%) males and 22 (14.8%) females. The median age was 60 years (range from 24 to 84 years old). Histological distribution was 30.2% squamous cell carcinoma (45 cases), 18.8% adenocarcinoma (28 cases), 38.5% small cell cancer (57 cases) and 12.8% others (19 cases). The majority of patients (65.1%) appeared stage III disease, 28.2% presented with stage IV disease, and 6.7% had stage I–II disease. Smokers accounted for 71.1% (106 cases) of the total. 25 (16.8%) patients were concomitant with chronic obstructive pulmonary disease. Notably, 13 (9.4%) patients had experienced surgery before radiation. Most of patients (89.9%) were treated with a combination of chemotherapy or target drugs therapy with radiotherapy. 84.6% patients received radiation with dose above 60Gy (median : 60Gy; range from 30 to 72Gy). The median values for MLD, V20 and V5 were 13.8Gy (range from 4.2 to 34.9), 22% (range from 5.0 to 35.5) and 58% (range from 15.0 to 92.7), respectively. The median follow-up time was 9 months (range from 3 to 24 months) after radiotherapy. Of the 149 patients, 10 cases (6.7%) developed RP of grade 3 or higher level, 57 (38.3%) developed RP of grade ≥ 2 RP, and 90 (62.7%) of grade 1 or lower level. The median time of grade ≥ 2 RP was 2.6 months (range from 1 to 6 months).

**Table 1:** Patient demographics and clinical information (*N* = 149)

Parameter (Variable)	All Patients (*n =* 149)	Grade 0-1RP (*n =* 92)	Grade ≥ 2RP (*n =* 57)
**Sex**			
Male	127 (85.2%)	82 (89.1%)	45 (78.9%)
Female	22 (14.8%)	10 (10.9%)	12 (21.1%)
KPS			
< 80	24 (16.1%)	13 (14.1%)	11 (19.3%)
≥ 80	125 (83.9%)	79 (85.9%)	46 (80.7%)
**Smoking History**			
No	43 (28.9%)	26 (28.3%)	17 (29.8%)
Yes	106 (71.1%)	66 (71.7%)	40 (70.2%)
COPD			
No	124 (83.2%)	80 (87.0%)	44 (77.2%)
Yes	25 (16.6%)	12 (13.0%)	13 (12.8%)
**Tumor Histology**			
Squamous cell carcinoma	45 (30.2%)	27 (29.3%)	18 (31.6%)
Adenocarcinoma	28 (18.8%)	20 (21.7%)	8 (14.0%)
Small cell lung cancer	57 (38.2%)	33 (35.9%)	24 (42.1%)
Others	19 (12.8%)	12 (13.1%)	7 (12.3%)
Stage			
I–II	10 (6.7%)	6 (6.5%)	4 (7.0%)
III	97 (65.1%)	59 (64.1%)	38 (66.7%)
IV	42 (28.2 %)	27 (29.4%)	15 (26.3%)
**Surgery**			
NO	136 (90.6%)	85 (92.4%)	51 (89.5%)
YES	13 (9.4%)	7 (7.6%)	6 (10.5%)
Chemotherapy			
No	15	7 (7.6%)	8 (14.0%)
Yes	134	85 (92.4%)	49 (86.0%)
**Median age(range)(y)**	60 (24–84)	59 (24–84)	62 (35–80)
**Median Radiation dose(range)(Gy)**	60 (30–72Gy)	60 (32–70)	61.6 (30–72)
**Bilateral Lung dose-volumehistogram**			
Median V5 (range)	58.0% (15.0–92.7%)	58.0% (15.0–92.7%)	58.5% (29.0–91.9%)
Median V20 (range)	22.0% (5.0–35.5%)	21.0% (5.0–29.6%)	23.5% (6.0–35.5%)
Median MLD (range)	13.8Gy (4.2–34.9Gy)	13.6Gy (5.3–22.4Gy)	14.2Gy (4.2–34.9Gy)

Abbreviations: RP = radiation pneumonitis; KPS = Karnofsky performance status; COPD = chronic obstructive pulmonary disease; V5 = volume of normal lung receiving 5Gy or more radiation; V20 = volume of normal lung receiving radiation of 20 Gy or more; MLD = mean lung dose.

Table 2 shows genotype distribution of the four detected SNPs. The rs11615 site (ERCC1, T354C) consisted of 57.0% cases of CC genotype, 40.3% cases of CT and 2.7% cases of TT genotypes respectively. Meanwhile, the rs3212986 (ERCC1, C1516A) locus, CC, CA and AA genotype carriers were 69 cases (45.6%), 65 cases (44.3%) and 15 cases (10.1%), respectively. Rs25487 (XRCC1, A1196G) comprised 58.4% GG, 34.9% GA and 6.7% AA genotype, respectively. In terms of rs13181 (ERCC2, A2251C), 124 (83.2%) were AA, and 25 (16.8%) were AC genotype carriers, but no any CC genotype were detected due to the extremely low frequency (almost lower than 1% of homozygous variants) of genotype in Chinese population, in consistent with the previous literature data [20–22]. All the other allele frequencies observed in the study were similar to those previously reported in Chinese population [23–26].

**Table 2:** Genotype frequency of gene polymorphisms in this study

Single-Nucleotide Polymorphism	All Patients (*n =* 149)	Grade 0-1RP (*n =* 92)	Grade ≥ 2RP (*n =* 57)
**ERCC1 rs11615 (T354C; Asn118Asn)**			
CC	85 (57.0%)	48 (52.2%)	37 (64.9%)
CT	59 (40.3%)	42 (45.6%)	17 (29.8%)
TT	5 (2.7%)	2 (2.2%)	3 (5.3%)
CT or TT	64 (43.0%)	44 (47.8%)	20 (35.1%)
**ERCC1 rs3212986 (C1516A; Gln504Lys)**			
CC	69 (46.3%)	42 (46.7%)	27 (47.4%)
CA	65 (43.6%)	39 (42.4%)	26 (45.6%)
AA	15 (10.1%)	11 (11.9%)	4 (7.0%)
CA or AA	80 (53.7%)	50 (53.3%)	30 (52.6%)
**ERCC2 rs13181 (A2251C; Lys751Gln)**			
AA	124 (83.2%)	78 (84.8%)	46 (80.7%)
AC	25 (16.8%)	14 (15.2%)	11 (19.3%)
CC	0	NC	NC
AC or CC	25 (16.8%)	14 (15.2%)	11 (19.3%)
**XRCC1 rs25487 (A1196G; Arg1196Gln)**			
GG	87 (58.4%)	51 (55.4%)	36 (63.2%)
GA	52 (34.9%)	34 (37.0%)	18 (31.6%)
AA	10 (6.7%)	7 (7.6%)	3 (5.2%)
GA or AA	62 (41.6%)	41 (44.6%)	21 (36.8%)

Abbreviations: ERCC1 =excision repair cross-complementing group 1; ERCC2 = excision repair cross-complementing group 2; XPD = xeroderma pigmentosum group D; XRCC1 = X-ray repair cross-complementing group 1; Ile = isoleucine; Val = valine; Ala = alanine; Gln = glutamine; Lys =lysine; Arg = arginine.

### Association of clinical variables and RP

We assessed the associations of developing grade ≥ 2 RP with parameters such a as therapy-related factors, including age, sex , races and Karnofsky Performance Score (KPS), tumor histology, disease stage, smoking history, COPD history, surgery history, usage of chemotherapy, radiation dose, V20, V5 and MLD by univariate and multivariate Cox regress analyses as presented in Table 3. We found that only V20 had statistically significant association with RP (grade ≥ 2) via both univariate and multivariate analyses (Table 3. bottom two lines), concordant with the results from a recent meta-analysis [27]. No other clinical factors were observed to be associated with RP risk in the study population.

**Table 3:** Association between clinical factors and RP (grade ≥ 2) incidence

Parameter	Patients	Number of	Percent of	Univariate Analysis			Multivariate Analysis^a^		
(Variable)		RPs (*n =* 57)	Patients with RP	HR	95% CI	*p*	AHR	95% CI	*p*
**Sex**									
Male	127	45	35.4%	1.0			1.0		
Female	22	12	54.5%	1.370	0.725–2.590	0.333	1.888	0.605–5.891	0.274
**Age (years)**									
< 60	71	24	33.8%	1.0			1.0		
≥ 60	78	33	42.3%	1.339	00.791–2.266	0.277	2.517	1.167–5.430	0.019
**KPS**									
< 80	24	11	45.8%	1.0			1.0		
≥ 80	125	46	36.8%	0.622	0.343–1.279	0.220	0.935	0.393–2.222	0.879
**Smoking Status**									
NO	43	17	39.5%	1.0			1.0		
YES	106	40	37.7%	1.092	0.619–1.927	0.761	1.690	0.622–4.590	0.304
**COPD**									
No	124	44	35.5%	1.0			1.0		
Yes	25	13	52.0%	1.621	0.872–3.013	0.127	1.043	0.400–2.724	0.931
**Tumor characteristics**									
**Histology**									
Squamous cell carcinoma	45	18	40.0%	1.0			1.0		
Adenocarcinoma	28	8	28.6%	0.643	0.279–1.478	0.298	0.800	0.264–2.420	0.800
Small cell lung cancer	57	24	42.1%	0.978	0.531–1.803	0.943	1.244	0.560–2.765	0.591
Others	19	7	36.8%	0.898	0.375–2.151	0.810	1.368	0.465–4.02	0.569
**Stage**									
I–II	10	4	40.0%	1.0			1.0		
III	97	38	39.2%	1.101	0.393–3.087	0.854	0.909	0.232–3.558	0.891
IV	42	15	35.7%	0.941	0.312–2.838	0.914	0.967	0.233–4.008	0.963
**Surgery**									
NO	136	51	37.5%	1.0			1.0		
YES	13	6	46.2%	1.193	0.512–2.781	0.683	3.048	0.959–9.686	0.059
**Chemotherapy**									
NO	15	8	53.3%	1.0			1.0		
YES	134	49	36.6%	0.729	0.345–1.541	0.408	0.549	0.204–1.475	0.234
**Radiation dose**									
< 60	23	7	30.4%	1.0			1.0		
≥ 60	126	50	39.7%	1.435	0.650–3.186	0.372	1.080	0.283–4.114	0.910
**Bilateral Lung dose-volume histogram**									
V5 < 58%	75	28	37.3%	1.0			1.0		
V5 ≥ 58%	74	29	39.2%	1.161	0.691–1.952	0.573	1.133	0.403–3.186	0.813
V20 < 20%	56	14	25.0%	1.0			1.0		
V20 ≥ 20%<25%	53	24	45.3%	2.043	1.057–3.950	0.034	2.971	1.085–8.133	0.034
V20 ≥ 25%	40	19	47.5%	2.182	1.094–4.354	0.027	5.810	1.391–24.27	0.016
MLD < 13.8Gy	74	24	32.4%	1.0			1.0		
MLD ≥ 13.8Gy	75	33	44.0%	1.372	0.811–2.321	0.239	1.605	0.666–3.872	0.292

Abbreviations: RP = radiation pneumonitis; HR = hazard ratio; KPS = Karnofsky performance status; COPD = chronic obstructive pulmonary disease; V5 = volume of normal lung receiving 5Gy or more radiation; V20 = volume of normal lung receiving 20 Gy or more radiation; MLD = mean lung dose.

a: Multivariate analyses were adjusted for all factors listed in this table.

### RP and genotype association

Table 4 shows the results of univariate and multivariate analyses of the correlation between the genetic polymorphisms and grade ≥ 2 RP via the Cox proportional hazards regression model. We observed that rs11615 SNP (ERCC1, T354C) was significantly associated with RP risk. Compared with CC genotype, the variant CT and TT genotypes were associated with decreased hazards of RP in the multivariate model (CT and TT vs. CC; HR = 0.465 & 0.517; 95% CI = 0.261–0.921 & 0.285–0.939; adjusted *P* = 0.027 & 0.030, respectively). Meanwhile, in case of rs25487 (XRCC1, A1196G), compared with the GG genotype, patients carrying GA or AA variants did not show significantly decreased RP risk (HR, 0.670, 95% CI, 0.367–1.225; adjusted *P* = 0.194; or HR, 0.649, 95% CI, 0.167–2.517; adjusted *P* = 0.531) respectively. When combination of GA or GG genotypes were considered, comparing with GG carriers, a slightly reduced but with no statistical significance risk was observed (HR, 0.667, 95% CI, 0.377–1.182; adjusted *P* = 0.166). There was no significant relationship between RP and other genetic polymorphisms were discovered.

**Table 4:** Associations between genotypes and grade ≥ 2 RP

Parameter	Patients	Number of	Percent of	Univariate Analysis			Multivariate Analysisa		
(Variable)		RPs (*n =* 57)	Patients with RP	HR	95% CI	*p*	AHR	95% CI	*p*
**ERCC1 rs11615 (T354C; Asn118Asn)^b^**									
CC	85	37	43.5%	1.0			1.0		
CT	59	17	28.8%	0.531	0.299–0.943	0.031	0.465	0.261–0.921	0.027
TT	5	3	60.0%	1.130	0.348–3.665	0.839	0.759	0.186–3.096	0.701
CT or TT	64	20	31.2%	0.576	0.334–0.994	0.048	0.517	0.285–0.939	0.030
**ERCC1 rs3212986 (C1516A; Gln504Lys)^b^**									
CC	69	27	39.1%	1.0			1.0		
AC	65	26	40.0%	1.049	0.612–1.798	0.861	0.906	0.481–1.708	0.761
CC	15	4	36.7%	0.639	0.224–1.827	0.403	0.774	0.251–2.388	0.656
AC or CC	80	30	37.5%	0.966	0.574–1.626	0.897	0.869	0.422–1.789	0.703
**ERCC2 (XPD) rs13181 (A2251C; Lys751Gln)^b^**									
AA	124	46	37.1%	1.0			1.0		
AC	25	11	44.0%	1.241	0.643–2.397	0.520	1.151	0.559–2.371	0.703
CC	0	NC	NC	NC	NC	NC	NC	NC	NC
AC or CC	25	11	44.0%	1.241	0.643–2.397	0.520	1.151	0.559–2.371	0.703
**XRCC1 rs25487 (A1196G; Gln399Arg)^b^**									
GG	87	36	41.4%	1.0			1.0		
GA	52	18	34.6%	0.732	0.416–1.290	0.281	0.670	0.367–1.225	0.194
AA	10	3	30.0%	0.653	0.201–2.122	0.479	0.649	0.167–2.517	0.531
GA or AA	62	21	33.9%	0.720	0.420–1.233	0.231	0.667	0.377–1.182	0.166

Abbreviations: RP = radiation pneumonitis; HR = hazard ratio; ERCC1 = excision repair cross-complementing group 1; ERCC2 = excision repair cross- complementing group 2; XPD = xeroderma pigmentosum group D; XRCC1 = X-ray repair cross- complementing group 1; Lys = lysine; Gln = glutamine; Arg = arginine; NC = not calculated.

a: Multivariate analyses in the table were adjusted for all factors listed in Table 3.

b: ERCC1 rs11615 or ERCC1 rs3212986 or ERCC2 rs13181 or XRCC1 rs25487 was independently entered into a multivariate model that was adjusted for all factors listed in Table 3.

**Figure 1:**
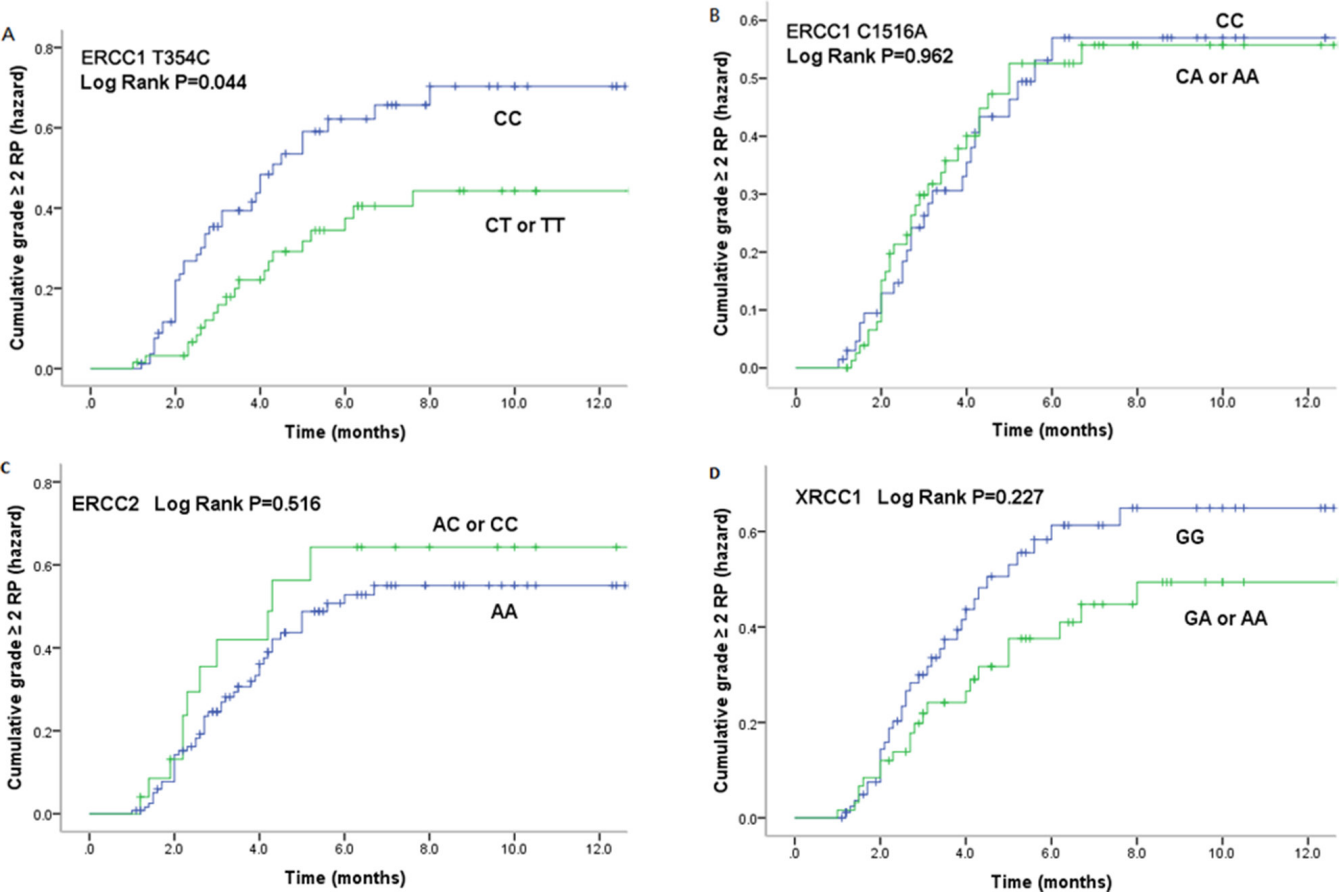
Cumulative probability of grade≥2RP as a function of time from the start of radiation therapy by genotypes (**A**). ERCC1 T354C; (**B**). ERCC1 C1516A; (**C**). ERCC2 A2251C; (**D**). XRCC1 A1196G . The CT/TT genotypes of ERCC1 354 were statistically significant associated with a lower incidence of RP compared with CC genotype.

Kaplan-Meier estimates indicated that patients with rs11615 CT/TT variants had significantly lower risk of grade ≥ 2 RP (*P* = 0.044) (Figure 1A). The cumulative incidence of grade ≥ 2 RP at 3 months for patients with either of these two variants and the CC genotype, which is more common in east Asian population, were 16.4% and 32.5% respectively, and those at 6 months were 33.3% and 48.1% respectively.

**Figure 2:**
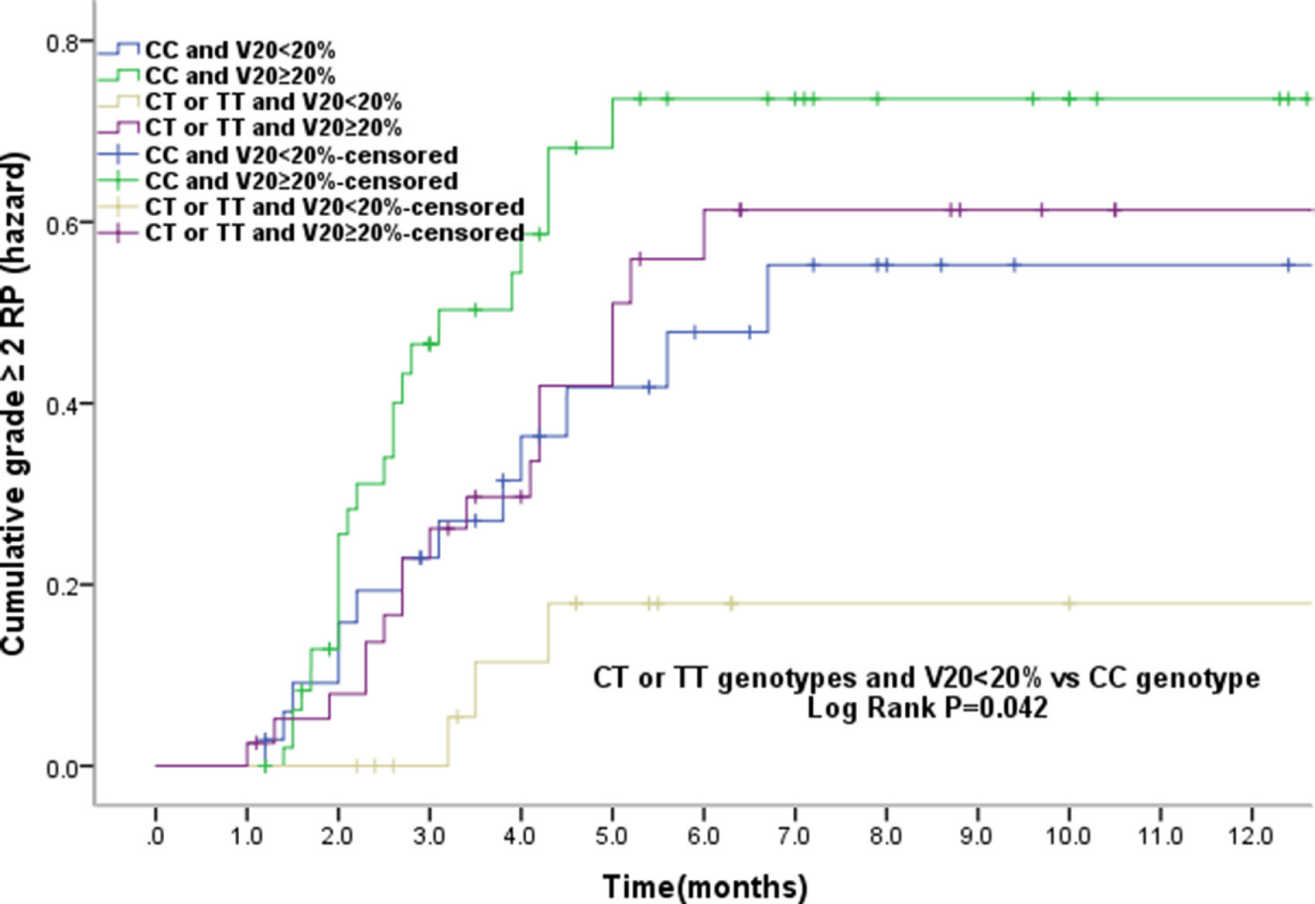
The effects of single nucleotide polymorphism at ERCC1 T354C with volume of normal lung receiving 20Gy or more radiation (V20) < 20% the cumulative incidence of grade ≥ 2RP Patients with the CT or TT genotypes of ERCC1 T354C and V20 < 20% had a statistically significant lower incidence of grade ≥ 2RP compared with CC genotype. The associations between ERCC1 T354C and the risk grade ≥ 2RP is independent of the V20.

In the study population, V20 was also observed as a significant risk factor of grade ≥ 2 RP. Therefore, we further analyzed the cumulative grade ≥ 2 RP events and the relationship between genotype and V20. The results showed that of all patients who received V20 < 20%, those with rs11615 CT/TT genotypes had a lower RP incidence than CC carriers (*P* = 0.042). However, in patients with V20 ≥ 20%, no difference was found to be of statistical significance (Figure 2), suggesting that as far as grade ≥ 2 RP risk concerned, the parameter V20 may be independent of genetic susceptibility.

## DISCUSSION

In the study, we assessed the associations of grade ≥ 2 RP events with clinical factors and genetic factors among lung cancer patients underwent IMRT. Our results showed that a genetic polymorphic site, rs11615, which is a functional variant of DNA repair gene ERCC1, may be an independent indicator for the RP risk. Therefore, taking genetic factors into account in clinical practice probably allow us to tailor customized radiotherapy protocols. NER is a versatile and important DNA repair system, and is responsible for repairing bulky DNA damage that includes drug-bound DNA adducts and UV-induced DNA changes [18, 28]. ERCC1, as the rate-limiting and highly conserved key enzyme in the multistep NER process, plays a critical role in the NER system. The function of ERCC1 is essential for maintaining the integrity and stability of genome, removing the copy mistakes, preventing gene mutation and tumorigenesis, and also influencing the level of whole BER repair activity [18]. Evidence suggests polymorphisms in ERCC1, may serve as a useful predictor for response to chemotherapies [28]. Previous reports illustrated that increased expression of ERCC1 might be related to better DNA repair capacity and a worse response to chemotherapy or vice versa [29, 30].

SNPs of ERCC1could affect mRNA expression and have attracted increasing interests as potential predictors of cancer therapy outcome and patient prognosis in lung cancer patients [18–19]. Two most common ERCC1 SNPs are rs11615 and rs3212986. Rs11615 is T→C substitution at exon 4 and this substitution is a synonymous silent polymorphism since resulting no amino acid residue change (Asn→Asn,) [31, 32]. However this variant does affect the transcription of ERCC1 and hence changes mRNA and protein levels. Therefore it also alters sensitivity to platinum-based therapeutics and may impact clinical outcome of patients receiving platinum-based chemotherapy [23, 28, 33]. In this study, we found that patients bearing rs11615 T (TT or CT) variant had a significantly reduced risk to develop RP compared to those carrying CC genotype (adjusted HR = 0.517; 95% CI, 0.285–0.939; adjusted *P* = 0.030), in consistent with the association in chemotherapeutic response previously reported. The possible reason may be that T allele carriers had elevated ERCC1 mRNA and thereby increased DNA repair activity. To our knowledge, this study is the first time that demonstrated that ERCC1 T354CSNP was significantly associated with RP risk among Chinese patients of ethnic Han with lung cancer administrated with radiotherapy, and implied this SNP as a promising biomarker for predicting normal tissue sensitivity to radiation. In addition, it is interesting that CT/TT genotypes carriers with V20 < 20% had lower risk of RP than those with CC genotype, but no significant genotype association was observed if V20 ≥ 20%. This finding suggests that genetic factors may have played an even more important role in RP susceptibility when a limited volume of lung was exposed to high dose of radiation. In terms of rs3212986 polymorphism, a C→A change in the 3’untranslated region, no functional difference had yet been reported [31]. It was implied to impact the ERCC1 mRNA stability but not influence the mRNA expression [34]. The polymorphism was found not to be related to the chemotherapeutic response but the outcome [20, 35]. The data in this study revealed that rs3212986 SNP had no statistical significant impact on RP risk, similar to that in chemotherapeutic response in previous report [20, 35].

As regarding to ERCC2, it is one of the most important DNA repair proteins and has dual functions in cells: nucleotide excision repair and cell cycle regulation through actions on the Cdk-activating kinase. It plays a crucial role in the NER pathway by recognizing and repairing a wide range of structurally unrelated lesions and oxidative damage. Several reports indicated that ERCC2 was also implicated in repairing ionizing radiation-induced DNA damage [36–37]. One common ERCC2 polymorphism is rs13181 variant, which changes codon at position 751 (a A→C substitution in exon 23) resulting Lys→Gln residue change. The C variant has been identified with a higher DNA adducts or lower DNA repair capacity. Only limited studies revealed no clear association of rs13181 polymorphism with acute or later side effects caused by radiation in breast cancer [38–39]. Instead, a recent study by Zhang *et al.* concluded that C variant was significantly associated with the risk of acute radiation-induced esophageal toxicity in lung cancer patients [40]. Based on the above facts, in this study we also explored whether rs13181SNP is related to RP risk in lung cancer, but no significant association was observed.

As for XRCC1, it is the first gene that was revealed to affect cell sensitivity to ionizing radiation, oxidative stress, and DNA alkylating agents [41]. The XRCC1 rs25487 variant is one of the most common polymorphism. Recently, Growing attentions have been paid to the association of rs25487 with the risk of normal tissue injuries after radiotherapy. However, the conclusion was ambiguous and even divergent results were reported [38, 39, 42, 43]. Two recent reports revealed the association of rs25487 with the risk of RP. A retrospective study reported by Yin *et al*, indicated that the G variant of rs25487, after adjusted for potential confounding factors, was associated with a reduced risk of RP in patients with non-small-cell lung cancer patients (HR = 0.48, *P* = 0.041) [42]. Then Kelsey *et al.* performed a prospective study to examine the correlation between XRCC1 and radiation-induced lung injuries in lung cancer patients receiving definitive radiotherapy. The result demonstrated that patients with the ancestral allele A were more radiosensitive to radiation (*P* = 0.01) [43]. In this study, we also found that patients carrying G variant had a lower risk of developing RP (HR, 0.653, 95% CI, 0.342–1.245), which is consistent with previous studies [42–43]. However as far as the population of this study, there was no statistical significance was reached, which agrees with the findings reported by Cheuk *et al* [44]. The possible reason for this discrepancy may be partly attributed to large ethnic frequency differences of rs25487 alleles.

In conclusion, the study provides the first clinical data that the rs11615 polymorphism in ERCC1 gene may have a predictive value for RP. The results suggest that in addition to the measures of clinical and radiation dosimetric factors, the naturally occurring genetic variants in ERCC1 gene may also be useful to predict the risk of RP. However, the results should be interpreted with caution in clinical practice since multi factors can contribute to the risk of RP, and moreover, further investigations are warranted to confirm these findings.

## MATERIALS AND METHODS

### Patients

In this prospective study, a group of 149 patients newly diagnosed with lung cancer were treated with definitive radiation between April 2014 and March 2016 at Chinese PLA General Hospital (Beijing, China). The eligible criteria were as follows: histologically or cytologically confirmed lung cancer, including non-small cell lung cancer or small cell lung cancer; no previous and co-existent thoracic radiotherapy; no severe radiotherapy contraindications; Karnofsky performance status (KPS) ≥ 60 scores. Symptom evaluation for each patient was required in advance. Patients’ characteristics and their outcomes were unknown to investigators performing genetic analyses. The results of genotyping were disclosed to clinical investigators after data analyses. This prospective research protocol was approved by the Internal Review Board of Chinese PLA General Hospital (Beijing, China), and conducted in agreement with the Helsinki Declaration. Written informed consent was obtained from all patients before undergoing radiotherapy.

### Radiation treatment

All patients received image-guided intensity-modulated radiation therapy (IMRT) with 6-MV photo beam. Patients underwent computed tomography (CT)-based treatment simulation in the supine position and immobilized in an upper body. Then, the images were transmitted to the Pinnacle planning system (version 9.2, Philips), and the target volume and critical normal organs were delineated. The total lung volume was defined as a total lung volume minus tumor. The IMRT plans were generated and optimized via the Pinnacle version 9.2 planning system, and the doses were calculated via the collapsed cone convolution dose algorithm. The plans for each patient were optimized by direct machine parameter optimization according to the target volume and the location of the organs at risk, and V20 was limited to 25–35%. The image-guided IMRT was performed via the two predefined linear accelerators (Elekta Synergy and Varian clinac ix). A total of 30–72Gy dose with the intention of radical or palliative treatment was applied once per day, five times per week. The baseline clinical characteristics and treatment details of the patients are shown in Table 1.

### Pulmonary toxicity assessment and follow-up

All patients included in the investigation were examined and evaluated prospectively by their radiation oncologists weekly during radiotherapy and 4–6 weeks after completion of treatment. Patients were then followed up every 3 months for the first 2 years and thereafter every 6 months. Additional visits were required if symptoms appeared. Radiographic examination by chest X-ray or computed tomography was performed at each follow-up visit after completion of radiotherapy. RP was evaluated by at least two radiation oncologists based on clinical symptoms and imaging information of each patient, and graded according to the Common Toxicity Criteria for Adverse Events (CTCAE) version 4 as follows: (1) Grade 0, no change; (2) Grade 1, asymptomatic and only observed by radiographic findings with no intervention indicated; (3) Grade 2, having symptoms limiting instrumental activities of daily living (ADL) and with medical intervention indicated; (4) Grade 3, having severe symptoms limiting self-care ADL and oxygen indicated; (5) Grade 4, having life-threatening respiratory compromise with urgent intervention indicated (e.g. tracheotomy or intubation); (6) Grade 5, resulting in death form severe RP. If the symptoms were present at baseline, worsening of symptoms of at least one grade was considered as RP. The following situations should be excluded when diagnosing the RP: (1) pulmonary infection; (2) thoracic disease progression (PD). The development of grade ≥ 2 RP was defined as the primary end point. The time to endpoint development was calculated from the start of radiation. Patients were censored at the time of last follow-up or death.

### DNA extraction and genotyping

The SNPs selected and their dbSNP ID (rs) were as follows: ERCC1 T354C (rs11615), ERCC1 C1516A (rs3212986), ERCC2 A2251C (rs13181) and XRCC1 1196A1196G (rs25487). Blood sample was collected from each patient before radiotherapy. Total genomic DNA from peripheral blood leukocytes was extracted with the Maxwell system (Promega, Madison, WI, USA). The SNP status was analyzed with the SurPlexTM-xTAG platform (Surexam, Guangzhou, China) as described by Zhu et al. including five major steps [45]. Generally, target genes were amplified with PCR and cleaned up with Exonuclease I and shrimp alkaline phosphatase (EXO-SAP) to remove excess nucleotides and primers. Then, the target genes were amplified again with 70 allele-specific primers linked to 70 universal tags. Using these tags, the amplified alleles were hybridized onto beads and analyzed with Luminex. The median fluorescence intensity was measured and analyzed. As to quality control, samples were randomly selected and sent to independent services for DNA sequencing analyses.

Multiplex PCR was performed in a 50 μl volume containing: 1× Ex Taq polymerase buffer, 2.5 mM MgCl_2_, 0.2 mM dNTPs, 0.2 mM of each primer, 1.0 U Ex Taq HS DNA polymerase (TaKaRa) and 10 ul supernatant of boiling DBS as template DNA. Thermo cycling was performed using 30 cycles of 95°C for 30 s, 56°C for 30 s, and 72°C for 30 s. The reaction was concluded with a final extension step of 72°C for 10 min and the product was kept at 4°C until use. A similar approach was used to enrich DNA fragments of *MTHFR* and *XPD* genes using 3 mM MgCl_2_. Exonuclease I and shrimp alkaline phosphatase (EXO-SAP) reaction was performed in a 25 μl volume containing: 1× SAP buffer, 1 U shrimp alkaline phosphatase (TaKaRa), 10 U exonuclease I (degrading any remaining PCR primers) (New England Biolabs, Ipswich, USA) and 7.5 μl PCR product. Samples were then incubated at 37°C for 30 min, followed by 20 min incubation at 80°C to inactivate the enzymes. Multiplex ASPE was carried out via 5 μl treated PCR product in a final volume of 20 μl. Each reaction consisted of SAP buffer, 1.25 mM MgCl_2_, 5 mM biotin-dCTP, 5 mM of dATP, dTTP and dGTP respectively; 0.75 U of Platinum Tsp; and 25 nM ASPE primer pool. The ASPE reactions were incubated at 96°C for 2 min and then subject to 40 cycles at 94°C for 30 s, 52°C for 1 min, and 74°C for 2 min.

### Statistical analysis

Patients were categorized according to their genotypes. Cox proportional hazard models were carried out to identify clinical variables and genotypes associated with the risk of RP on univariate and multivariate analyses. Kaplan-Meier analysis was performed to assess the effects of different genotypes on cumulative probability of RP and comparisons were made with the log-rank test. All statistical tests were 2-sided, and *P* values < 0.05 were of statistically significance. Data analyses was performed via the IBM SPSS Statistics platform (Version 20, SPSS Science, IL, USA).
